# Pulmonary Artery Stump Thrombosis: To Treat or Not to Treat? The Question Is Still Open. Description of a Case and Review of the Literature

**DOI:** 10.3389/fcvm.2021.714826

**Published:** 2021-10-04

**Authors:** Antonio Mirijello, Mariateresa Santoliquido, Pamela Piscitelli, Cristina Borelli, Gaetano Serviddio, Anna Simeone, Elvira Grandone, Salvatore De Cosmo

**Affiliations:** ^1^Internal Medicine Unit, Department of Medical Sciences, Fondazione IRCCS Casa Sollievo Della Sofferenza, San Giovanni Rotondo, Italy; ^2^Geriatrics Residency School, Department of Medical and Surgical Sciences, University of Foggia, Foggia, Italy; ^3^Radiology Unit, Fondazione IRCCS Casa Sollievo Della Sofferenza, San Giovanni Rotondo, Italy; ^4^Thrombosis and Haemostasis Research Unit, Fondazione IRCCS Casa Sollievo Della Sofferenza, San Giovanni Rotondo, Italy

**Keywords:** anticoagulation, pneumonectomy, pulmonary embolism, thrombosis, pulmonary artery stump

## Abstract

Pulmonary artery stump thrombosis (PAST) represents a possible complication after lung surgery. We report the case of a 59-year-old man who presented with dyspnoea about 4 years after right pneumonectomy due to squamous cell lung cancer. A CT-scan showed the presence of pulmonary artery stump thrombosis. Although there was no evidence of pulmonary embolism, given the clinical features and radiological shape of the thrombus, anticoagulation treatment with low-molecular-weight heparin was started with improvement of symptoms. The patient was discharged on anticoagulant treatment and a pulmonary CT-scan performed 4 months later showed an almost complete resolution of the PAST. Pathophysiological mechanisms of PAST are still unknown, although several hypotheses have been proposed. However, the decision to treat PAST with anticoagulants is still controversial. A review of literature will be provided in order to discuss risk factors, possible etiologies and to highlight clinical and radiological characteristics that could suggest to treat this condition, in particular when there is an increased risk of complications.

## Introduction

Pulmonary artery stump thrombosis (PAST) following pulmonary resection for lung cancer is a possible complication after lobectomy (1) or pneumonectomy (2), with an incidence of 12% (2) after this latter. Generally, PAST occurs early after surgery (3); nevertheless, a delayed presentation has been described, although sporadically (4–10). Pathophysiological mechanisms of PAST are not fully understood, although several hypotheses have been formulated, such as endothelial damage during surgery, hypercoagulability and blood flow stasis in the vascular stump. PAST is generally asymptomatic and incidentally detected at follow-up CT-scans; moreover, it is usually harmless (11). However, in a minority of cases, it could be complicated with pulmonary embolism to the contralateral lung (4), pulmonary hypertension (6) and death (8, 12–14). At present, the optimal treatment of PAST is still matter of debate.

Here we describe the late-occurrence of PAST in a patient treated with right pneumonectomy due to squamous cell lung carcinoma. Clinical features, possible causes and risk factors will be reported. Moreover, a review of the literature will be provided in order to discuss gray areas concerning treatment options and the choice to treat this specific case.

## Case Presentation

In October 2020, a 59-year-old man was admitted to our Internal Medicine inpatients unit because of the persistence for about 2 weeks of dyspnoea, fatigue, and weight loss. The patient also reported right hypochondrium pain and loss of appetite. Past medical history was relevant for hypertension, type 2 diabetes and alcohol abuse (reported alcohol consumption: 3–5 drinks per day from the age of sixteen). In 2016 he was diagnosed with squamous cell lung carcinoma (stage T4N3M0) and treated with neoadjuvant polychemotherapy (cisplatine + vinorelbin) followed by right pneumonectomy. Despite a history of cancer, he was still an active smoker. His home therapy consisted of insulin and acetylsalicylic acid 100 mg/day.

**Figure 1:**
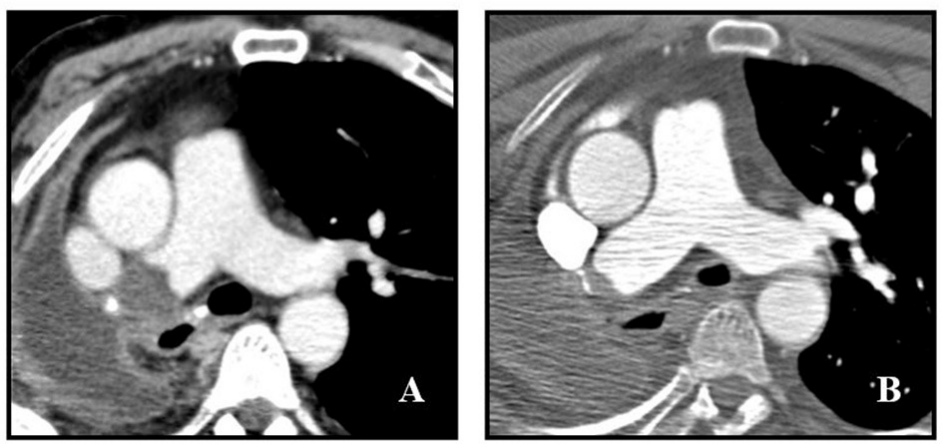
Post-pneumonectomy thrombosis of right pulmonary artery stump. **(A)** Initial contrast-enhanced CT scan shows a large endoluminal filling defect in the right pulmonary artery stump. **(B)** Follow up contrast-enhanced CT scan obtained 4 months after **(A)** shows marked reduction of thrombus size with evidence of a small residual intraluminal defect adjacent to surgical clips.

At admission, blood pressure was 150/75 mmHg, heart rate 92 bpm, oxygen saturation 94% in room air, respiratory rate 20/min, body temperature was 36°C. Physical examination was non-significant apart from hepatomegaly. Results of laboratory tests, including blood gas analysis, at admission are shown in Supplementary Table 1. In particular, acute phase reactants (e.g., fibrinogen, C-reactive protein, ferritin), transaminases, cholestasis enzymes and D-dimer were altered. Hepatitis B and C markers were negative. Sars-CoV-2 nasopharyngeal swab was negative. Chest X-ray showed opacification and volume loss of right hemi-thorax with consensual mediastinal shift, according to history of previous pneumonectomy; no signs of pulmonary consolidation in the left lung. Abdominal US-scan showed hyper-echogenicity of the liver compatible with steatosis and/or fibrosis and biliary sludge. No significant kidneys or spleen abnormalities nor ascites were found. The Esophago-Gastro-Duodenoscopy detected a grade B reflux disease (LA classification), congestive gastropathy and erosive bulb duodenitis. Basing on history, clinical features and Wells' score (0 points) (15), PE was unlikely. An echocardiography showed a normal left ventricle ejection fraction, no right ventricle overload nor pulmonary hypertension. To rule out cancer recurrence, total body CT-scan with contrast injection was performed. Chest CT images showed a pulmonary thrombus within the right main artery stump, not present 1 year earlier (Figure 1A). Doppler US-scan of lower limbs was normal. Anticoagulant treatment with enoxaparin 100 ui/kg/bid was started, together with proton pump inhibitor (PPI). Tests for inherited and acquired thrombophilia were negative as well as antibodies against Sars-Cov-2. Patient's symptoms gradually improved and he was discharged 7 days after PAST diagnosis with anticoagulant prescription. Contrast-enhanced CT scan performed 4 months later demonstrated an almost complete resolution of right pulmonary thrombosis (Figure 1B).

## Discussion

PAST is a possible complication after lung surgery (2), generally occurring within 12 months (early PAST), even if a delayed presentation has also been described (late PAST) (4–10). Usually it is asymptomatic and incidentally detected during a follow-up CT-scan (3). However, English literature reports anecdotal cases of patients complaining of dyspnoea finally diagnosed with PAST (Table 1). At present, risk factors and mechanisms at the basis of this thrombotic event are still poorly understood. In a retrospective analysis of 648 oncologic surgeries for primary lung cancer, 25 (3.8%) PAST were found (3). Among them, elderly age, advanced cancer stage and neo-adjuvant chemotherapy could represent risk factors for its development (3). Regarding pathophysiological mechanisms for stump thrombosis, Virchow's triad has been indicated as one of the possible causes (3). On this connection, three processes could be hypothesized:

1. *Endothelial injury during surgery*. Complex surgeries and vascular manipulations increase the risk of endothelial damage, reducing the production of fibrinolytic activators with a consequent pro-coagulant state (16). For this reason, the technique with continuous ligature of the stump has been considered more appropriated than the transfixation one, obtaining a regular stump which decreases the probability of PAST. However, this mechanism could be mainly responsible for early PAST.
2. *Blood flow stasis of the vascular stump*. There is a correlation between stump's length and changes in blood flow dynamics (e.g., blood flow turbulence) (11). Basing on the retrospective revision of chest CT-scans of patients treated with pneumonectomy, Kim and co-workers (2) evidenced that the stump is longer after a right pneumonectomy than after a left pneumonectomy, and the thrombus was more frequently detected in the right (23.3%) than in the left (4.6%) stump. On the contrary Kwek and colleagues (11) found an almost equal incidence of thrombi between right and left stumps. Concerning lobectomy, left-sided thrombi were more common than right-sided one (3).
3. *Hypercoagulability*. Increased platelet count and fibrinogen level at 7th and 14th day after lung surgery were observed as factors associated with higher thrombotic activity (17). In addition, other factors such as smoking (18), active cancer (19), and sepsis (20) are known to increase blood coagulability. To date, an association between PAST and inherited/acquired thrombophilia has never been described.

**Table 1:** Cases of PAST reported in the English literature.

**References**	**Gender**	**Age**	**Type of lung resection**	**Side**	**Symptoms**	**Timing**	**Lower limb Doppler US**	**Treatment**	**Resolution**	**Complications**	**Death**
Barbetakis et al. (22)	Male	59	Lobectomy	Right	No	6 months	Negative	Heparin, then OAC for 6 months	Yes	No	No
Sato et al. (4)	Male	73	Pneumonectomy	Left	Chest discomfort	8 years	N.a.	Heparin	Yes	Controlateral pulmonary embolism (anticoagulant therapy was discontinued)	No
Thomas et al. (6)	Male	51	Pneumonectomy	Right	Astenia back pain dyspnoea	10 years	Deep venous thrombosis	Heparin	No	Multiple pulmonary emboli and pulmonary hypertension	Probably
Chuang et al. (12) n1	Female	29	Pneumonectomy	Right	Dyspnoea, tachycardia	8 months	N.a.	No	No	Infarction left lower lobe	Yes
Chuang et al. (12) n2	Male	65	Pneumonectomy	Right	Dyspnoea	24 h	N.a.	No	No	Emboli left lower lobe and lingular arteries	Yes
Gorospe Sarasúa (13)	Male	71	Pneumonectomy	Right	Dyspnea	5 month	/	Heparin	No	Death	Yes
Akcam et al. (9)	Male	73	Pneumonectomy	Left	No	3 years	Negative	Heparin, then warfarin	Yes	No	No
Joshi et al. (8)	Male	68	Pneumonectomy	Right	Pleuric chest pain	10 years	Negative	Heparin, then warfarin	No	Yes	Yes
Viola et al. (10)	Female	76	Lobectomy	Right	Dyspnoea	6 years		Rivaroxaban	No	/	No
Sawalha and Mador (5)	Male	67	Lobectomy	Right	No	2 years	Negative	Coumadin for 3 months	Yes	No	No
Kotoulas and Lachanis (23)	Male	53	Pneumonectomy	Right	No	3 months	Negative	Acenocumarol	Yes	No	No
Yoon et al. (7)	Female	75	Pneumonectomy	Right	Dyspnea	10 years	N.a.	Warfarin	Yes	Multiple small thrombi in left pulmonary artery	No
Gorospe et al. (14)	NA	NA	Lobectomy	Left	No	1 year	N.a.	Heparin	Yes	No	No
Dury et al. (24) (3 cases)	NA	NA	Pneumonectomy	/	/	/	/	No	/	No	No
Wechsler et al. (25)	Female	72	Lobectomy	Right	Shortness of breath, fatigue	11 months	Negative	No	No	No	No

The need for anticoagulation in patients with PAST is still matter of debate. According to literature, early PAST is more likely to resolve spontaneously, regardless from anticoagulation, while late PAST usually shows a poor rate of resolution (21).

Among the 17 case reports of PAST reported in the English literature, seven of them were late PASTs (Table 1); all received anticoagulant therapy, the majority of which resolved.

Moreover, anticoagulation could be started according to the morphology of PAST at CT scan. Convex-shaped and floating PAST are considered more acute and at high-risk of embolization to the contralateral lung or of growth. On the contrary, concave-shaped thrombi are considered at lower risk of embolization and more “stable” (11). According to some authors, anticoagulation should be considered for convex thrombi or for a newly occurring PAST in the context of declining pulmonary status (8). Indeed, it is important to understand if PAST represents an *in situ* thrombosis, a cancer recurrence inside the vascular stump or an embolus from deep vein thrombosis (2); all of these could be indications for treatment. However, the incidence of PAST-associated PE is low (5). According to the available reports, one patient showed contralateral pulmonary embolism (4) and another one showed multiple pulmonary emboli with pulmonary hypertension (6); both of them had been treated, but just one solved (Table 1).

We reported the case of a patient complaining of dyspnoea, fatigue and abdominal pain, and weight loss. Since the presenting symptomatology was not specific for any particular disease, several examinations were performed. Chest X-ray did not show abnormalities in the left lung and, according to Wells' score, pre-test probability of PE was low. Abdominal symptoms and weight loss were consistent with chronic alcoholic liver disease, gastro-oesophagitis and duodenitis. Respiratory symptom was not justified by blood gas analysis, not showing respiratory failure. Finally, chest CT-scan evidenced the presence of PAST. According to CT-scan (Figure 1A), this was a newly evidenced late-occurring PAST with a convex-shaped thrombus in the right pulmonary artery stump with no evidence of PE. These characteristics, in conjunction with the presence of symptoms, surrounding inflammatory state, history of active smoking and previous chemotherapy led us to the decision to start anticoagulation. The CT-scan performed 4 months later found an almost complete resolution of the clot (Figure 1B).

## Conclusions

PAST represents a possible complication after lung surgery, in particular after right pneumonectomy. Local and systemic factors seem to be involved in its pathophysiology, although the exact mechanisms are not completely understood. Generally, PAST represents an occasional finding at follow-up CT scan and it is asymptomatic; sometimes, patients could complain of dyspnoea, fatigue and chest discomfort. The choice to prescribe anticoagulants should be based on the risk of complications, such as contralateral pulmonary embolism, worsening lung function and death. At present, literature data on factors potentially favoring embolization are few and decision-making algorithms are lacking. It is conceivable that the evidence of convex-shaped thrombi and patient's thrombotic risk could represent the most important factors suggesting the need for anticoagulation. In any case, the choice for optimal treatment duration and follow-up should be evaluated case by case.

## Ethics Statement

Written informed consent was obtained from the patient for the publication of any potentially identifiable images or data included in this article.

## Author Contributions

AM, MS, PP, and EG managed the patient during hospitalization. AM, MS, and PP thought about the study rationale. CB and AS reviewed radiological images. GS and SD reviewed collected data. AM, MS, PP, EG, and SD wrote the first draft. All Authors read, had the possibility to modify and approved the final draft of the paper.

## Conflict of Interest

The authors declare that the research was conducted in the absence of any commercial or financial relationships that could be construed as a potential conflict of interest.

## Publisher's Note

All claims expressed in this article are solely those of the authors and do not necessarily represent those of their affiliated organizations, or those of the publisher, the editors and the reviewers. Any product that may be evaluated in this article, or claim that may be made by its manufacturer, is not guaranteed or endorsed by the publisher.
