# Supplementary material for: Pulmonary Artery Stump Thrombosis: To Treat or Not to Treat? The Question Is Still Open. Description of a Case and Review of the Literature
Source: Front Cardiovasc Med. 2021 Oct 4;8:714826. doi: 10.3389/fcvm.2021.714826 (PMC8520941; doi:10.3389/fcvm.2021.714826)
Supplement: Supplementary file 1 [file Table_1.DOC]

**Supplementary Table 1 – Results of lab tests at admission (T0)**

| **Blood parameters** | **Values** | **Normal range** |
| --- | --- | --- |
| HB – g/dl | 15.6 | 13-17 |
| WBC – ×1000/uL | 7.39 | 4.3-10.8 |
| PLT – ×1000/uL | 257.00 | 130.00-400.00 |
| Sodium (Na) – mmol/L | 136.0 | 136.0-145.0 |
| Potassium (K) – mmol/L | 4.0 | 3.5-5.0 |
| Chloride (Cl) – mmol/L | 102.0 | 98.0-107.0 |
| Creatinine – mg/dL | 0.8 | 0.69-1.30 |
| Albumin – g/dL | 3.10 | 3.50-5.50 |
| Calcium – mmol/l | **1.06** | 1.12-1.32 |
| Total bilirubin – mg/dl | **2.30** | 0.20-1.00 |
| AST – U/L | **153.0** | 8.00-30.00 |
| ALT – U/L | **90.0** | 13.00-57.00 |
| ALK-P – UI/L | **275** | 45-117 |
| y-GT – UI/L | **4167.00** | 5.00-85.00 |
| PT – % | 94 | 70-130 |
| aPTT ratio | 0.93 | < 1.2 |
| INR – ratio | 1.04 | 0.80-1.20 |
| PTT – sec | 24.4 | 20.0-32.0 |
| Fibrinogen – mg/dl | **403** | 150-400 |
| D-dimer – ng/ml | **574.00** | 0.00-500.00 |
| CRP – mg/dL | **0.71** | < 0.290 |
| LAC | 1.13 | <1.20 |
| aCL IgM – U/mL | 4.60 | <15 |
| aCL IgG – U/mL | <1.6 | < 15 |
| Prothrombin mutation (G20210A) | ABSENT | ABSENT |
| V Leiden factor mutation (G1691A) | ABSENT | ABSENT |
| ANA | **1/160** | NEGATIVE |
| ENA | NEGATIVE | NEGATIVE |
| AAC: AMA | NEGATIVE | NEGATIVE |
| AAC: ASMA | NEGATIVE | NEGATIVE |
| HbsAg | NEGATIVE | NEGATIVE |
| Anti HCV | NEGATIVE | NEGATIVE |
| Ferritin – ng/ml | **1700.0** | 26.0 – 388 |
| SARS-CoV-2 serology | NEGATIVE | NEGATIVE |
| pH (ABG) | 7.38 | 7.35 – 7.45 |
| pO2 – mmHg (ABG) | 70.8 | 80.0 – 100.0 |
| pCO2 – mmHg (ABG) | 44.8 | 35.0 – 45.0 |
| Anion Gap - mmol/l (ABG) | 10.6 | 8.00 – 16.00 |
| HCO3^-^  - mmol/l (ABG) | 25.9 | 22.0 – 26.0 |

HB = haemoglobin, WBC = white blood count, PLT = Platelet count; AST = Aspartate aminotransferase, ALT = alalnine aminotransferase; ALK-P = alkaline phosphatase, gGT = gamma-glutamyltranspeptidase; PT = Prothrombin time; INR = international normalized ratio; PTT = Partial thromboplastin time; CRP = C reactive protein; ESR = Erythrocyte sedimentation rate; ANA = Antinuclear antibodies; ENA= extractable nuclear antigens; aCL = anti-cardiolipin antibodies; LAC = lupus anticoagulant; anti-smooth muscle antibodies (ASMA), anti-mitochondrial antibodies (AMA); ABG = arterial blood gas analysis
